# A prediction and interpretation machine learning framework of mortality risk among severe infection patients with pseudomonas aeruginosa

**DOI:** 10.3389/fmed.2022.942356

**Published:** 2022-07-25

**Authors:** Chen Cui, Fei Mu, Meng Tang, Rui Lin, Mingming Wang, Xian Zhao, Yue Guan, Jingwen Wang

**Affiliations:** Department of Pharmacy, Xijing Hospital, Fourth Military Medical University, Xi'an, China

**Keywords:** machine learning, interpretation, stratification analysis, Pseudomonas aeruginosa, severe infection, risk factors

## Abstract

Pseudomonas aeruginosa is a ubiquitous opportunistic bacterial pathogen, which is a leading cause of nosocomial pneumonia. Early identification of the risk factors is urgently needed for severe infection patients with P. aeruginosa. However, no detailed relevant investigation based on machine learning has been reported, and little research has focused on exploring relationships between key risk clinical variables and clinical outcome of patients. In this study, we collected 571 severe infections with P. aeruginosa patients admitted to the Xijing Hospital of the Fourth Military Medical University from January 2010 to July 2021. Basic clinical information, clinical signs and symptoms, laboratory indicators, bacterial culture, and drug related were recorded. Machine learning algorithm of XGBoost was applied to build a model for predicting mortality risk of P. aeruginosa infection in severe patients. The performance of XGBoost model (AUROC = 0.94 ± 0.01, AUPRC = 0.94 ± 0.03) was greater than the performance of support vector machine (AUROC = 0.90 ± 0.03, AUPRC = 0.91 ± 0.02) and random forest (AUROC = 0.93 ± 0.03, AUPRC = 0.89 ± 0.04). This study also aimed to interpret the model and to explore the impact of clinical variables. The interpretation analysis highlighted the effects of age, high-alert drugs, and the number of drug varieties. Further stratification clarified the necessity of different treatment for severe infection for different populations.

## Introduction

Pseudomonas aeruginosa (P. aeruginosa), a ubiquitous Gram-negative pathogen, can colonize almost any part of the human body (1). More than 50% of severe acute and chronic hospital-acquired infections are caused by P. aeruginosa (2), such as ventilator-associated pneumonia and catheter infections in immunocompromised patients (3–5). It contributes to mortality rates as high as 13.5% in ventilation-associated pneumonia caused by P. aeruginosa (6). The most common cause of death from cystic fibrosis is P. aeruginosa lung infections (7).

Pseudomonas aeruginosa infection with diverse pathological background exerts a heavy health burden for modern society. Thus, there is an urgent need to identify the mortality risk factors of infection for severe infection patients with P. aeruginosa early. A retrospective study has shown that APACHE II score and septic shock are critical factors for mortality in P. aeruginosa bacteremia, and combination therapy does not significantly reduce overall 14-day mortality (8). Several other studies have analyzed the risk factors for mortality of P. aeruginosa using logistic regression, such as age, sex, ICU admission, glucocorticoid use, inappropriate treatment regimens, mechanical ventilators, the use of a central venous catheter, and a higher APACHE II score (8–10). In a multi-center study, risk factors for mortality of community-acquired P. aeruginosa included previous pseudomonas infection/colonization, tracheostomy, bronchiectasis, invasive respiration and/or vasopressor therapy (IRVS), and very severe chronic obstructive pulmonary disease (COPD) (11). Using of previous antibiotic and ICU admission is important risk factors for drug-resistant P. aeruginosa (12), which increases the number of days in hospital stays and all-cause mortality in hospitalized patients significantly (13). Most of studies above used traditional logistic regression to predict the risk factors of P. aeruginosa infection, and there was no research for identification of the mortality risk prediction of P. aeruginosa infection in severe patients.

Machine learning is a data-driven computing method, which does a lot of work based on big data. While machine learning has been demonstrated in a few different fields, it has only recently been gaining popularity in the field of medicine. Compared to logistic regression, machine learning methods are often more comprehensive, accurate, and rapid in clinical risk prediction (14). Various machine learning methods have been widely used in constructing prediction models of disease risk, such as gastrointestinal bleeding risk assessment, prediction of mortality in intensive care units, and sepsis-associated thrombocytopenia (15–17). Ma et al. used an unsupervised learning algorithm to classify septic shock into five phenotypes, investigate the associated risk factors, and determine the best treatment strategy for these phenotypes (18). However, there has not yet been a machine learning method for the mortality risk of severe infection patients with P. aeruginosa.

In this study, we proposed a mortality risk prediction framework for severe infection patients with P. aeruginosa infection based on machine learning. Our framework focused on decision support and model interpretation. Based on XGBoost algorithm and electronic medical records (EMR) data, we built a machine learning model with good predictive performance using grid searching and cross-validation (19). Furthermore, the SHapley Additive exPlanation (SHAP) values were used to explain the prediction model from a global perspective for overcoming the shortcomings of machine learning models (20). It has the advantage of providing more details about the relationship between predictive variables and outcomes, and describing in detail the relationship between clinical factors and risks. The interpretative analysis revealed key clinical features of the risk of mortality P. aeruginosa infection in severe patients. Finally, we conducted a stratified analysis of patients from three aspects: infection site, advanced age, and the number of intravenous drug varieties. The results have some implications for P. aeruginosa clinical practice. Our study enables accurate predictions of the risk of mortality P. aeruginosa infection in severe patients, as well as interpretation of key variables that can support clinical decision making more accurately and effectively.

## Materials and methods

### Patient selection

The study was conducted at the Xijing Hospital of the Fourth Military Medical University, and a total of 571 patients with severe infections were included in the study between January 2010 and July 2021. There were 338 patients in the death group and 233 patients in the control group. Our study was approved by the domestic ethics committee with the approval number KY20212130-C-1. This study is a retrospective, observational study design that does not require informed consent. The collected research data were de-identified and analyzed anonymously.

### Data collection

Data collected using EMR at the First Affiliated Hospital of Fourth Military Medical University: basic information: age, sex, etc.; drug related: number of drug varieties, number of antibiotics drugs varieties, high-alert medication, etc.; clinical signs and symptoms: headache, cough, temperature, etc.; laboratory indicators: white blood cell count, absolute neutrophil value, etc.; bacterial culture: blood culture, urine culture, etc. All data collected are provided in the Supplementary Section (Supplementary Table 1). Here, high-alert medication refers to drugs that may cause serious injury or death to patients due to improper use of medication errors (17). According to the severity of adverse consequences that may be caused by their clinical use, high-alert medication is divided into 3 grades: A, B, and C. For the specific classification of high-alert medication, please refer to the recommended list of high-alert medication in China recommended by the Chinese Pharmaceutical Association (https://www.cpa.org.cn/index.php?do=info&cid=75676) and the management of high-alert medication in Xijing Hospital of the Fourth Military Medical University. The details of high-alert medication can be found in the Supplementary Section (Supplementary Table 2).

### Inclusion criteria and exclusion criteria

Inclusion criteria: From January 2010 to July 2021, hospitalized patients with severe infection who associated with P. aeruginosa infection; Diagnosis of severe infection with P. aeruginosa, severe infection was defined as requiring at least 3 days of intravenous antibiotic therapy and at least 3 days of hospitalization after the diagnosis of confirmed infection. The ICD code for the diagnosis of severe infection in this study is shown in Supplementary Table 3. The P. aeruginosa infection was defined by combining the patient's clinical symptoms, signs, laboratory indicators, microbial culture, imageology, etc. Culture specimens of microorganisms come from different sites of infection, such as blood culture, urine culture, and sputum culture. The result of the patient's treatment was death or recovery.

Exclusion criteria: Non-P. aeruginosa infection; Patients with incomplete data and medical record information (the missing value of laboratory indicators exceeds 50%, incomplete medical history, no medication records); Some comorbidities such as autoimmune diseases (systemic lupus erythematosus, ANCN-associated vasculitis, rheumatoid arthritis, etc.,), malignant tumors (stomach cancer, ovarian cancer, lung cancer, etc.,) were excluded; Suspected contaminated specimens (the same sample culture of 3 or more pathogenic bacteria); Non-infected or colonized patient, such as the patient's clinical symptoms, signs, laboratory indicators, imageology were not abnormal; Hospitalization for less than 3 days.

### Preprocessing and imputation of clinical variables

All the clinical variables we collected could be divided into numerical and categorical variables according to clinical significance, and longitudinal and non-longitudinal variables according to whether repeated monitoring occurred during admission. Then, the categorical variables were converted into one-hot vectors. For clinical longitudinal variables, we extracted the maximum increase and maximum decrease during hospitalization for each variable. For laboratory longitudinal variables, we extracted the slope of all laboratory variables over time, the maximum increase and decrease during hospitalization. Finally, we got 91 variables in total (including derived variables). A detailed description and classification of all variables can be found in the Supplementary Section (Supplementary Table 1).

Outliers were detected using the interquartile range (IQR). As a threshold, the 2 times of IQR were used, and points exceeding this threshold (the upper quartile + 2 times of IQR, or the lower quartile – 2 times of IQR) were defined as outliers. Data points out of the valid value threshold were identified as outliers. The excluded outliers were modified as the nearest threshold.

Variables which had more than 50% missing values were deleted, while variables which had less than 20% missing values were replaced by the median values. Multivariate imputation by chained equations (MICE) was used to impute missing values while loss rates of variables were between 20 and 50%.

Finally, the *z*-score normalization was only performed for the all continuous values used by Support Vector Machine (SVM) (21). Since tree-based models such as XGBoost did not require standardization, the *z*-score normalization step was omitted when interpreting XGBoost, LightGBM (22), CatBoost (23), and Random Forests (RF) (24).

### Model algorithm

The XGBoost is a scalable end-to-end tree boosting system, which implements machine learning algorithms in a gradient enhancement framework that is efficient, flexible, and portable. It could be used for handling sparse data, and solving many data science problems quickly and accurately. The XGBoost has been widely used by data scientists to obtain state-of-the-art results in many machine learning challenges. The equations were as follow:


(1) $$\begin{array}{l} L\left(\emptyset \right)=\overset{n}{\underset{i}{\sum }}l\left({ŷ}_{i},y_{i}\right)+\overset{k}{\underset{j}{\sum }}\Omega \left(f_{j}\right) \end{array}$$

Here, *l* is a loss function that measures the differences between the prediction ŷ_*i*_and the target *y*_*i*_. The Ω penalizes the complexity of the model.

In order to minimize the L, the function could be write as:


(2) $$\begin{array}{l} {\tilde{L}}^{\left(t\right)}=\overset{n}{\underset{i=1}{\sum }}\left[g_{i}f_{t}\left(X_{i}\right)+\frac{1}{2}h_{i}f_{t}^{2}\left(X_{i}\right)\right]+\Omega \left(f_{t}\right) \end{array}$$

(3) $$\begin{array}{l} g_{i}=\partial_{{ŷ}^{\left(t-1\right)}}l\left(y_{i},{ŷ}^{\left(t-1\right)}\right) \end{array}$$

(4) $$\begin{array}{l} h_{i}=\partial_{{ŷ}^{\left(t-1\right)}}^{2}l\left(y_{i},{ŷ}^{\left(t-1\right)}\right) \end{array}$$

Here, all XGBoost models were implemented by using XGBoost (version 1.5.1). All codes were implemented using Python 3.7.9.

### Method comparison

In order to evaluate the performance of our model, we compared the XGBoost with LightGBM, CatBoost, SVM, and RF methods. All models have been optimized by grid searching to adjust hyperparameters. The detailed hyperparameters of XGBoost were described in Section 3.2. We selected the best model for predicting mortality risk for patients with severe P. aeruginosa infection.

The different parameters of LightGBM, CatBoost, SVM, and RF are summarized in Supplementary Section (Supplementary Tables 4–7). LightGBM and CatBoost were implemented by lightgbm 3.3.2 and catboost 1.0.6 in Python 3.7.9. The SVM and RF models were implemented by using scikit-learn. All code was implemented using Python 3.7.9.

### Evaluation metrics

The performance of the machine learning classifier was assessed using accuracy (ACC), receiver operator characteristics (ROC) curve, precision recall (PR) curve, area under the receiver operator characteristics curve (AUROC), and area under the precision recall curve (AUPRC), as defined by the following metrics:


(5) $$\begin{array}{l} ACC=\frac{TP+TN}{TP+TN+FP+FN} \end{array}$$

(6) $$\begin{array}{l} Recall=True\;Positive\;Rate=\frac{TP}{TP+FN} \end{array}$$

(7) $$\begin{array}{l} Precision=\frac{TP}{TP+FP} \end{array}$$

(8) $$\begin{array}{l} False\;Positive\;Rate=\frac{FP}{TN+FP} \end{array}$$

where TP, TN, FP, and FN refer to true positives, true negatives, false positives, and false negatives, respectively. Here, a “positive” label means that the outcome indicator of sample is death.

### Interpretation algorithm

In order to interpret the prediction results of XGBoost, Shapley additive explanations values were introduced, which unify Shapley regression values (20), Shapley sampling values, local interpretable model-agnostic explanations (LIME) (25), and other three existing additive feature attribution methods (DeepLIFT) (26), layer-wise relevance propagation (27), and quantitative input influence. Shapley values were defined as a class of additive feature attribution methods, which have an explanation model that is a liner function of binary variables as follow:


(9) $$g\left(z\text{'}\right)=\emptyset_{0}+\sum_{i=1}^{M}\emptyset_{i}z_{i}\text{'}$$

Where *z*′∈{0, 1}^*M*^, *M* is the number of simplified input feature, and ∅_*i*_∈ℝ. ∅_0_ is the constant of the interpretation model, ∅_*i*_ is the predicted mean value of all training samples, and is the attribution value of each feature.

### Statistical analysis

In this paper, two independent-sample *t*-tests were used for the statistical analysis. A *p*-value of less than 0.05 was considered significant. All statistical analyses were performed using Scipy 1.7.2.

## Results

### General information

**Figure 1:**
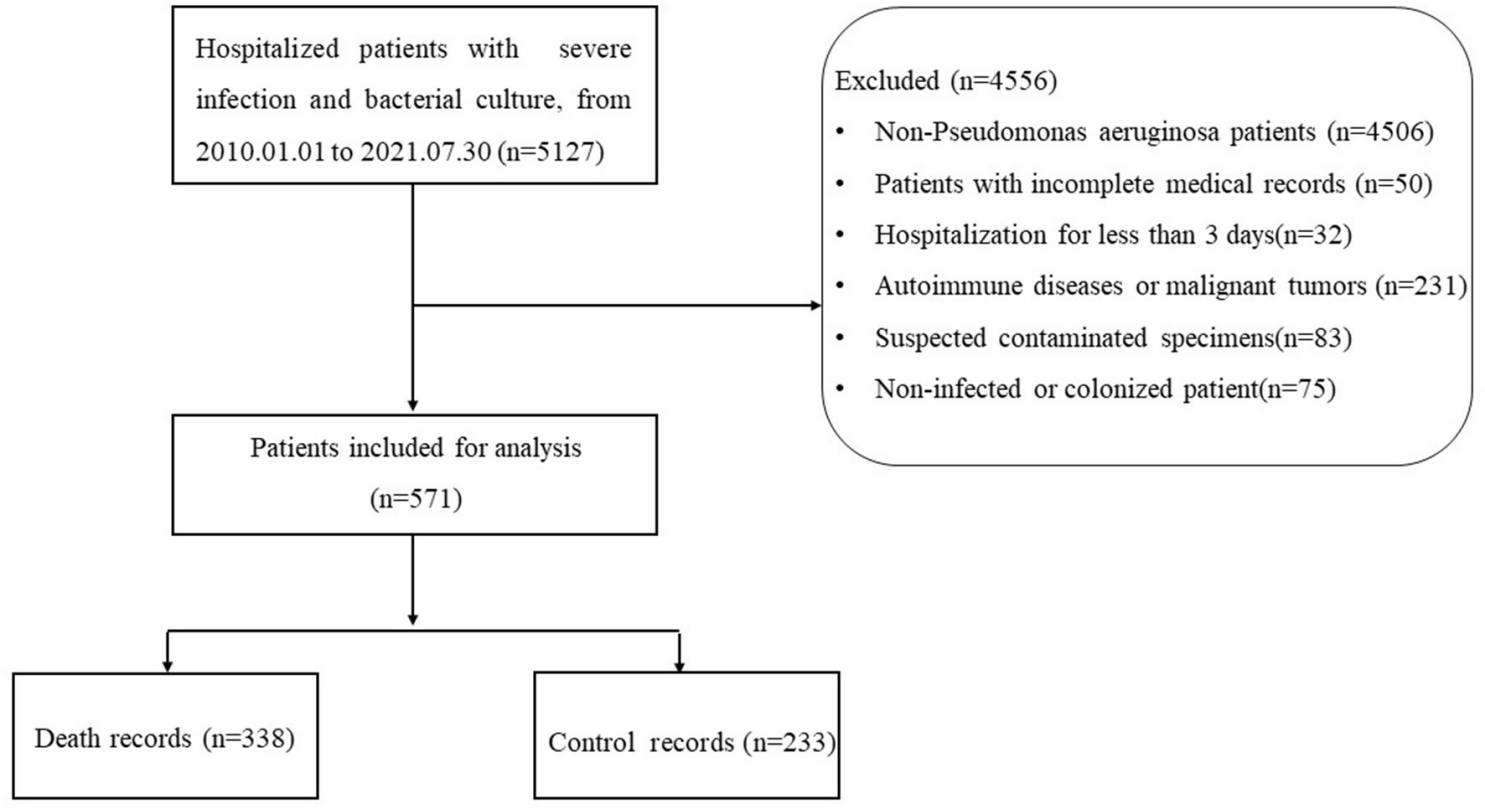
Flow chart of the study.

A total of 571 hospitalized patients infected with P. aeruginosa were included in this study. The flow chart of this study is shown in Figure 1. In terms of the source of infection, pulmonary infections accounted for the highest percentage of 455 cases (80%), followed by bloodstream infections with 57 cases (10%) and skin and soft tissue infection with 54 cases (9%). A detailed description of the clinical characteristics of the whole cohort is provided in Table 1.

**Table 1:** Characteristics of patients at baseline and clinical outcomes.

**Categories**	**Variables**	**Total (*****n*** = **571)**
Basic information	Age (years) [median (IQR)]	64 (47–81)
	Male [No. (%)]	428 (74.86%)
	Hosp (days) [median (IQR)]	23 (13–40)
	Drug Allergy [No. (%)]	69 (12%)
	Smoking [No. (%)]	105 (18%)
	Alcohol User [No. (%)]	55 (10%)
Drug related	Number of Drug Varieties [median (IQR)]	52 (39-66)
	Number of Intravenous Drugs Varieties [median (IQR)]	7 (4-10)
Clinical signs and symptoms	Headache [No. (%)]	91 (16%)
	Cough [No. (%)]	365 (64%)
	Expectoration [No. (%)]	322 (56%)
	Sore Throat [No. (%)]	15 (3%)
	Hemoptysis [No. (%)]	7 (1%)
	Dyspnea [No. (%)]	149 (26%)
	Vomiting [No. (%)]	187 (33%)
	Diarrhea [No. (%)]	76 (13%)
	Lymphadenopathy [No. (%)]	14 (2%)
	Drainage [No. (%)]	222 (39%)
	Tracheotomy [No. (%)]	104 (18%)
	Endotracheal Intubation [No. (%)]	150 (26%)
	Central Venous Catheter [No. (%)]	43 (8%)
	Indwelling Catheter [No. (%)]	302 (53%)
	PICC Catheter [No. (%)]	141 (25%)
	Temperature (°C) [median (IQR)]	36.9 (36.5–37.6)
	Respiratory Rate (min^−1^) [median (IQR)]	21.0 (19.0–25.0)
	Heart Rate (min^−1^) [median (IQR)]	89.0 (78.0–105.0)
	DBP (mmHg) [median (IQR)]	68.0 (60.0–76.0)
	SBP (mmHg) [median (IQR)]	116.0 (102.0–129.0)
Bacterial culture	Blood [No. (%)]	57 (10%)
	Urine [No. (%)]	16 (3%)
	Phlegm [No. (%)]	455 (80%)
	Secretions [No. (%)]	54 (9%)
	Cerebrospinal Fluid [No. (%)]	7 (1%)
	Feces [No. (%)]	0 (0%)
	Number of Concurrent Infection [No. (%)]	399 (70%)
Laboratory Indicators	WBC(s×10^9^/L) [median (IQR)]	10.08 (6.9–14.39)
	NEUT# (×10^9^/L) [median (IQR)]	7.96 (5.12–11.82)
	NEUT% [median (IQR)]	0.83 (0.74–0.89)
	RBC (×10^12^/L) [median (IQR)]	3.19 (2.77–3.66)
	PLA (×10^9^/L) [median (IQR)]	166.0 (91.0–258.0)
	HGB (g/L) [median (IQR)]	95.0 (84.0–110.0)
	ALT (IU/L) [median (IQR)]	29.0 (17.0–57.0)
	AST (IU/L) [median (IQR)]	31.0 (20.0–55.0)
	DBIL (μmol/L) [median (IQR)]	8.4 (4.6–16.0)
	CREA (μmol/L) [median (IQR)]	78.0 (59.0–115.0)
	Urea (mmol/L) [median (IQR)]	8.87 (5.7–15.0)
	ALB (g/L) [median (IQR)]	31.6 (28.5–34.8)
	SAA (mg/L) [median (IQR)]	202.0 (72.1–421.0)
	ESR (mm/h) [median (IQR)]	56.0 (26.25–84.5)
	CRP (mg/L) [median (IQR)]	60.5 (25.25–116.15)
	IL-6 (pg/mL) [median (IQR)]	56.96 (25.03–139.2)
	PCT (ng/mL) [median (IQR)]	0.95 (0.31–3.42)

*NEUT# represents the neutrophil count*.

### Model optimization and performance

To optimize the XGBoost model, the dataset was divided into five sets. One of the five sets was selected as test set, the rest four sets were selected as training set. We explored different hyperparameters through a grid search, such as the maximum depth, the number of estimators, and learning rate. We considered the maximum depth with 2, 4, 8, 16, and 32, the number of estimators with 5, 10, 15, 20, 25, and 30, and the learning rate with 0.01, 0.05, 0.1, 0.2, 0.3, and 0.5. The best models with different hyperparameters were selected according to the mean performance based on cross validation.

**Figure 2:**
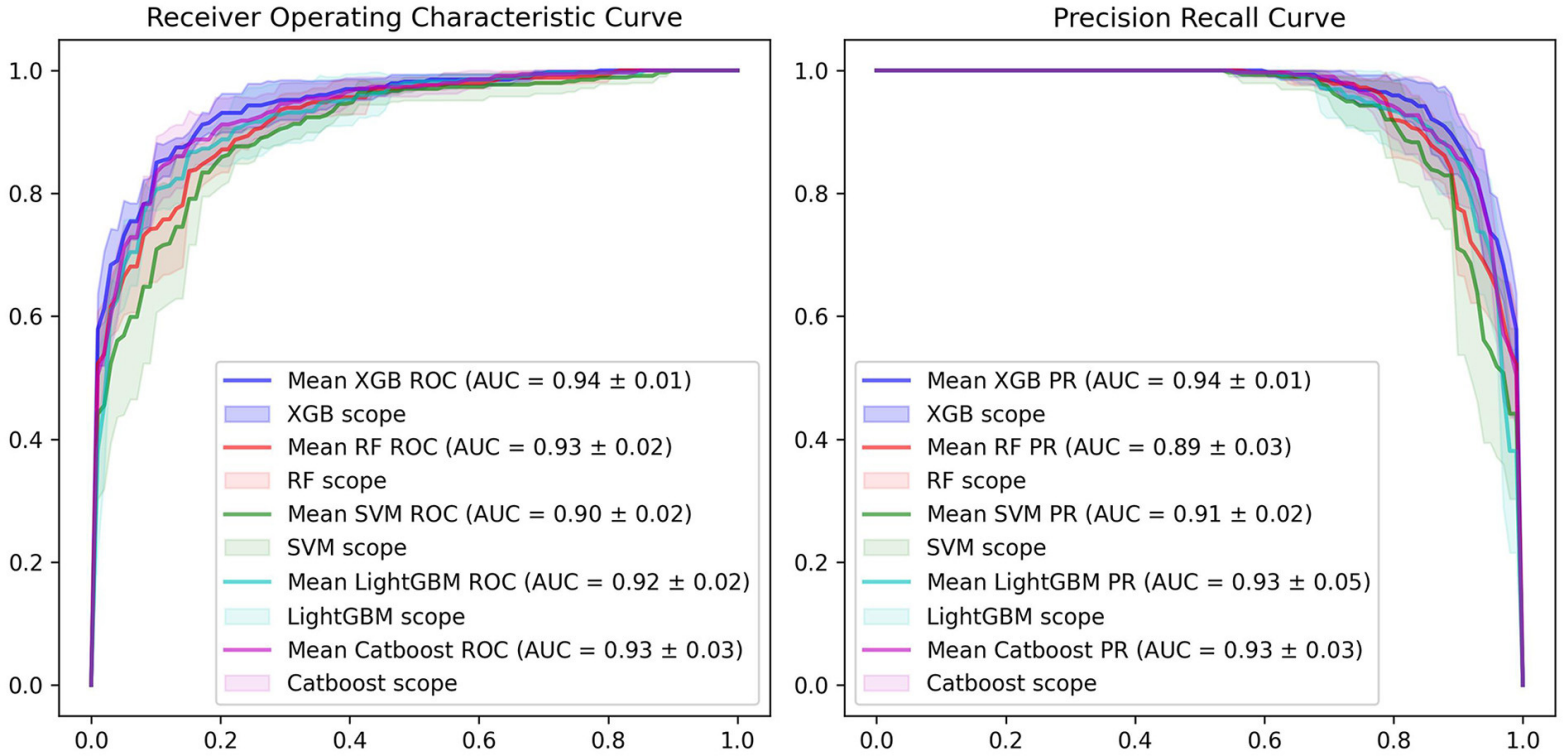
Receiver operator characteristics (ROC) curve and precision recall (PR) curve of five machine learning models.

The ROC curve and PR curve of three machine learning models are shown in Figure 2. The AUC of 5-fold cross validations were between 0.90 and 0.96 and PR of 5-fold cross validations was between 0.91 and 0.97. The ACC, AUROC, and AUPRC of mean performance of 5-fold cross validation were displayed in Table 2. The results shown that XGBoost had better prediction ability than other methods.

**Table 2:** Methods comparison based on AUROC and AUPRC.

**Method**	**ACC**	**AUROC**	**AUPRC**
XGBoost	0.88 ± 0.02	0.94 ± 0.01	0.94 ± 0.03
LightGBM	0.86 ± 0.05	0.92 ± 0.02	0.93 ± 0.05
CatBoost	0.86 ± 0.02	0.93 ± 0.03	0.93 ± 0.03
Random Forest	0.86 ± 0.03	0.93 ± 0.03	0.89 ± 0.04
Support Vector Machine	0.84 ± 0.03	0.90 ± 0.03	0.91 ± 0.02

### Model interpretation

Although the XGBoost model can achieve good predictions performance, the lacking of interpretation limits the application in clinical practice. To facilitate interpretation of the prediction model, an artificial intelligence SHAP values for global model interpretation were introduced (20). Compared with traditional feature importance methods (such as decision tree weights importance), SHAP values have better consistency and can show the positive or negative relationship of each predictive variable with respect to the target.

#### Importance of clinical variables

**Figure 3:**
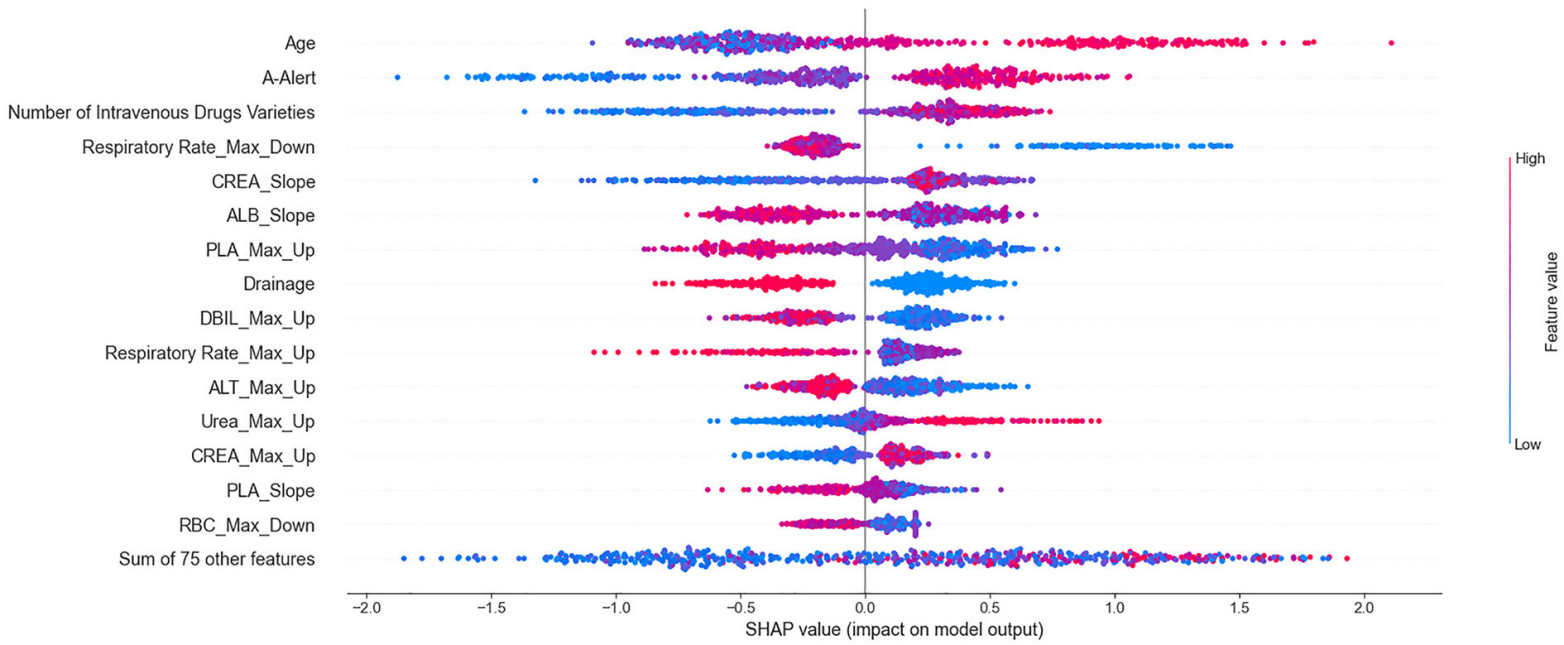
Summarize bee swarm plots for top 15 clinical variables of SHAP values. In a bee swarm plot, each point corresponding to a sample of single P. aeruginosa infected patient of data set. The position of each point on the horizontal axis indicated the effect of that feature on the model prediction, and the color of a point reflected the eigenvalue of the case. For binary variables (such as drainage or not), red dots and bule dots correspond to 1 and 0 respectively. For numerical variables (such as age), the color of dots represented high and low values, respectively. Overlapping points that fall in the same horizontal position will be scattered vertically to show the density.

According to the importance and impacts of variables on model prediction, a bee swarm plot was formed for each feature. As shown in Figure 3, a series bee swarm plots were listed in their order of importance.

We found that older (red) patients had a higher risk of mortality than younger (blue) patients (large on the horizontal). Similarly, patients who used more types of high-alert medications and more types of intravenous drugs had higher risk of mortality than those who used fewer types. The lower the maximum decrease in respiratory rate (significantly lower than normal), the higher the risk of mortality. In addition, patients who underwent drainage (red) had a lower risk of mortality than patients who did not undergo drainage (blue). It is important to emphasize that all effects only describe the behavior of the model and are not causality in the real world.

#### Detailed dependencies of variables

**Figure 4:**
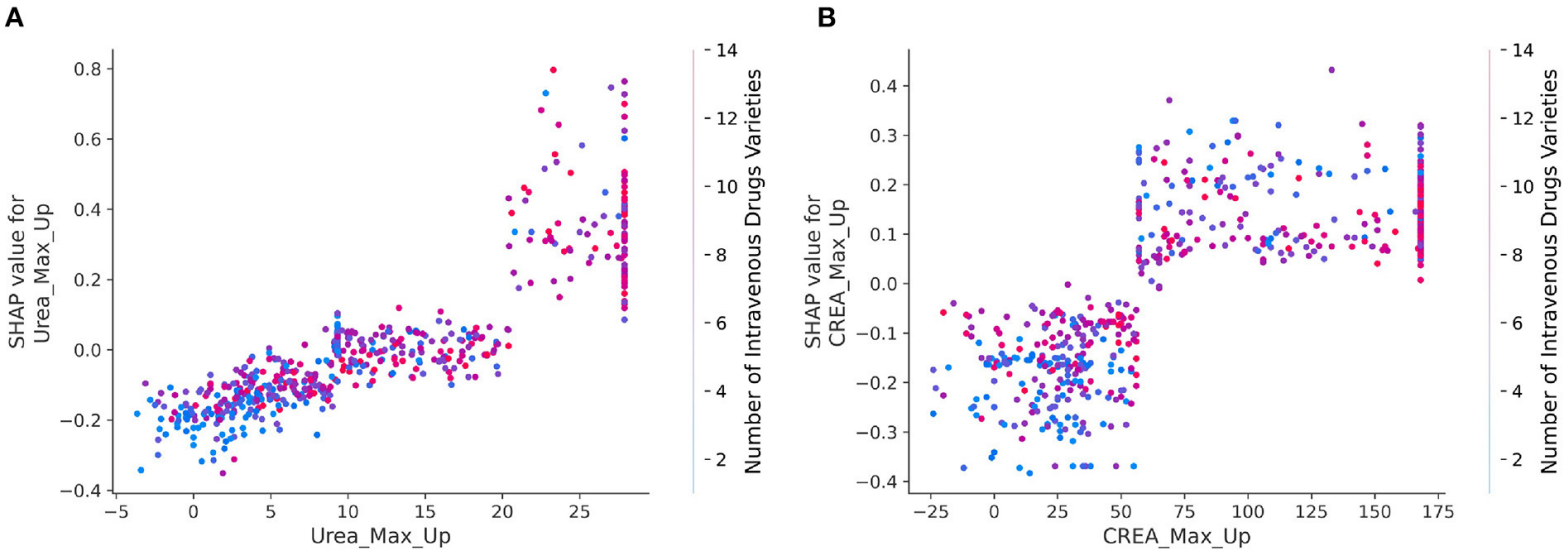
**(A,B)** SHAP dependence plots for interaction of crucial clinical variables. The x axis represents the eigenvalue of the axis title, and the y axis indicates the corresponding SHAP value, representing the contribution of this feature to prediction results of model. The color of every dot reflects the eigenvalues of right axis title. The larger the value of the x-coordinate of the sample point, the variable of x-axis is more large. The larger the value of the y coordinate of the sample point, the greater risk of mortality of the sample point, and the redder the color of the sample point, the higher the value of the right index.

To further elucidate the detailed relationship between mortality risk and clinical variables, SHAP interaction values were used to reveal the dependencies relationships based on the key feature of importance the bee swarm plots. Here, each point corresponds to a sample of infected patients, and each scatter plot shows the effect of features on SHAP interaction values. The results were shown in SHAP dependence plots (Figure 4). By analyzing the dependencies factors, it was found that the risk of mortality was significantly higher in patients with higher maximum increases in urea and creatinine when the number of intravenous drugs was higher.

### Evaluation on different pathogens infection

In addition to P. aeruginosa, Klebsiella pneumoniae (K. pneumoniae) is major hospital-acquired pathogen, causing pneumonia, urinary tract infection, intra-abdominal infection, and bacteremia in immunocompromised patients (28).

Here, we build a clinical dataset of K. pneumoniae infections as an external validation for testing and discussing the generalization performance of our model. The hyperparameters of model were obtained from the best performance in Section 3.2. Five sub-models trained on the 5-fold cross validation were used in the external validation set. The average performance of each sub-model on these external test sets is shown in Table 3. We can find that the performance of model on K. pneumoniae still had some degree of predictive ability, but it is a little worse than prediction for infection patients with P. aeruginosa. It suggested that our prediction model had some capacity for prediction on different pathogens infection.

**Table 3:** Performance of model on external validation sets.

**Infection**	**ACC**	**AUROC**	**AUPRC**
P. aeruginosa	0.88 ± 0.02	0.94 ± 0.01	0.94 ± 0.03
K. pneumoniae	0.85 ± 0.04	0.91 ± 0.03	0.92 ± 0.05

### Stratification analysis

#### Stratified analysis of infection sites

**Figure 5:**
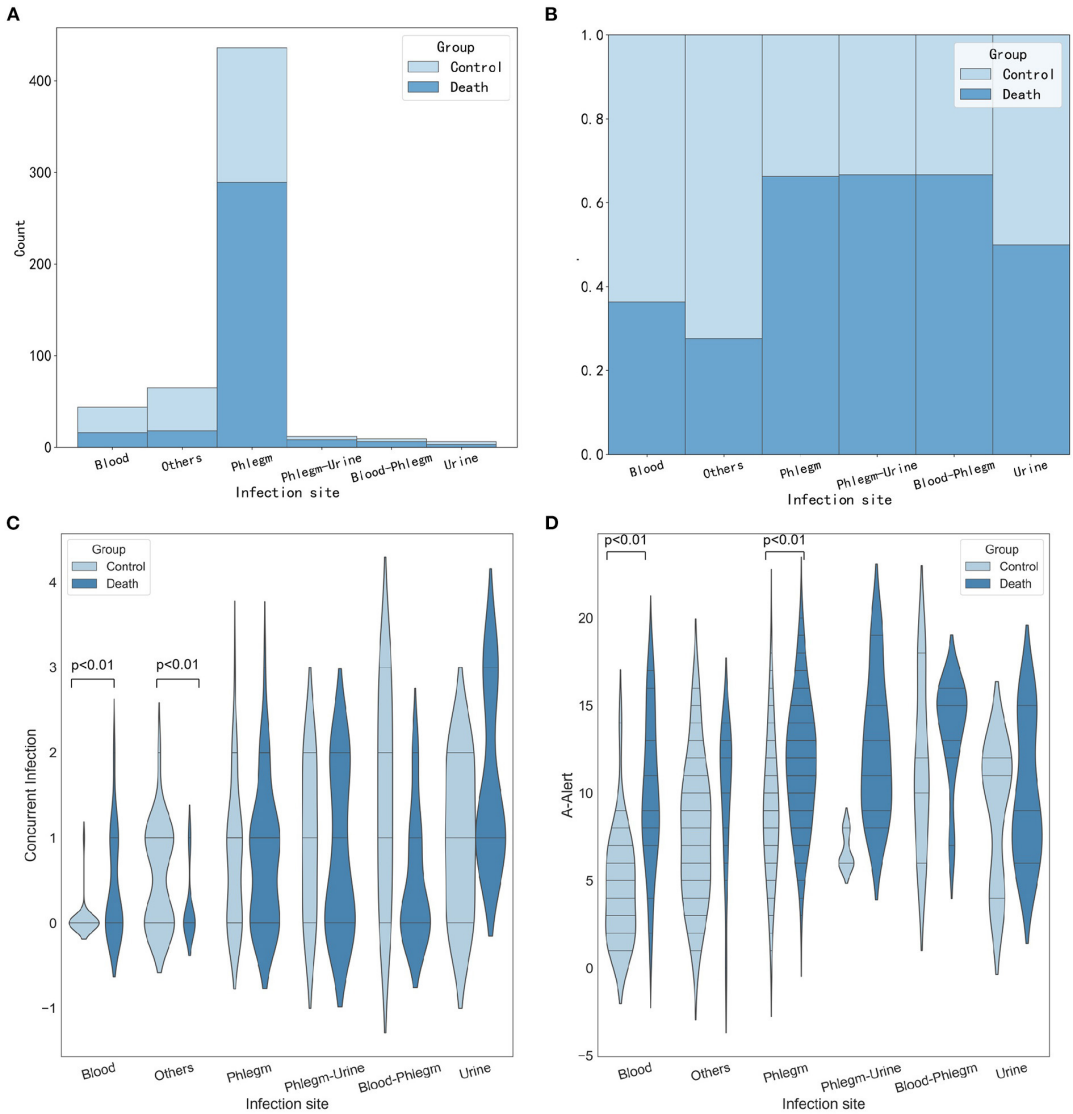
Stratified analysis of infection sites. **(A)** Histogram showing the number of P. aeruginosa cultured at different infection sites. **(B)** Histogram showing the proportion of P. aeruginosa cultured at different infection sites. **(C)** Violin plots showing the number of concurrent infections between different infection sites. **(D)** Violin plots showing the number of A-Alert drugs between different infection sites.

Figures 5A,B shows the number and proportion of P. aeruginosa cultured at different infection sites. The percentage of P. aeruginosa cultured in phlegm, others, and blood was higher. Here, others refer to infection sites except blood, urine, phlegm, secretions, cerebrospinal fluid, and feces. Compared with the control group, more P. aeruginosa was cultured in phlegm of patients in the death group. And the proportion of P. aeruginosa cultured in sputum and urine, sputum and blood at the same time was significantly higher than the death group. At the same time, we analyzed the association between the infection site and the number of concurrent infections. Figure 5C suggests that there was a statistically significant difference between the number of co-infections in the death group and the control group (*p* < 0.01), and the death group was often accompanied by 0–3 co-infections, when infection site was blood. The association between the infection site and the number of high warning drugs used was also analyzed. Figure 5D shows that when P. aeruginosa was detected in sputum culture or blood culture, the number of A-Alert drug use was more in the death group than in the control group, and the difference was significant (all *p* < 0.01).

#### Stratified analysis of age

**Figure 6:**
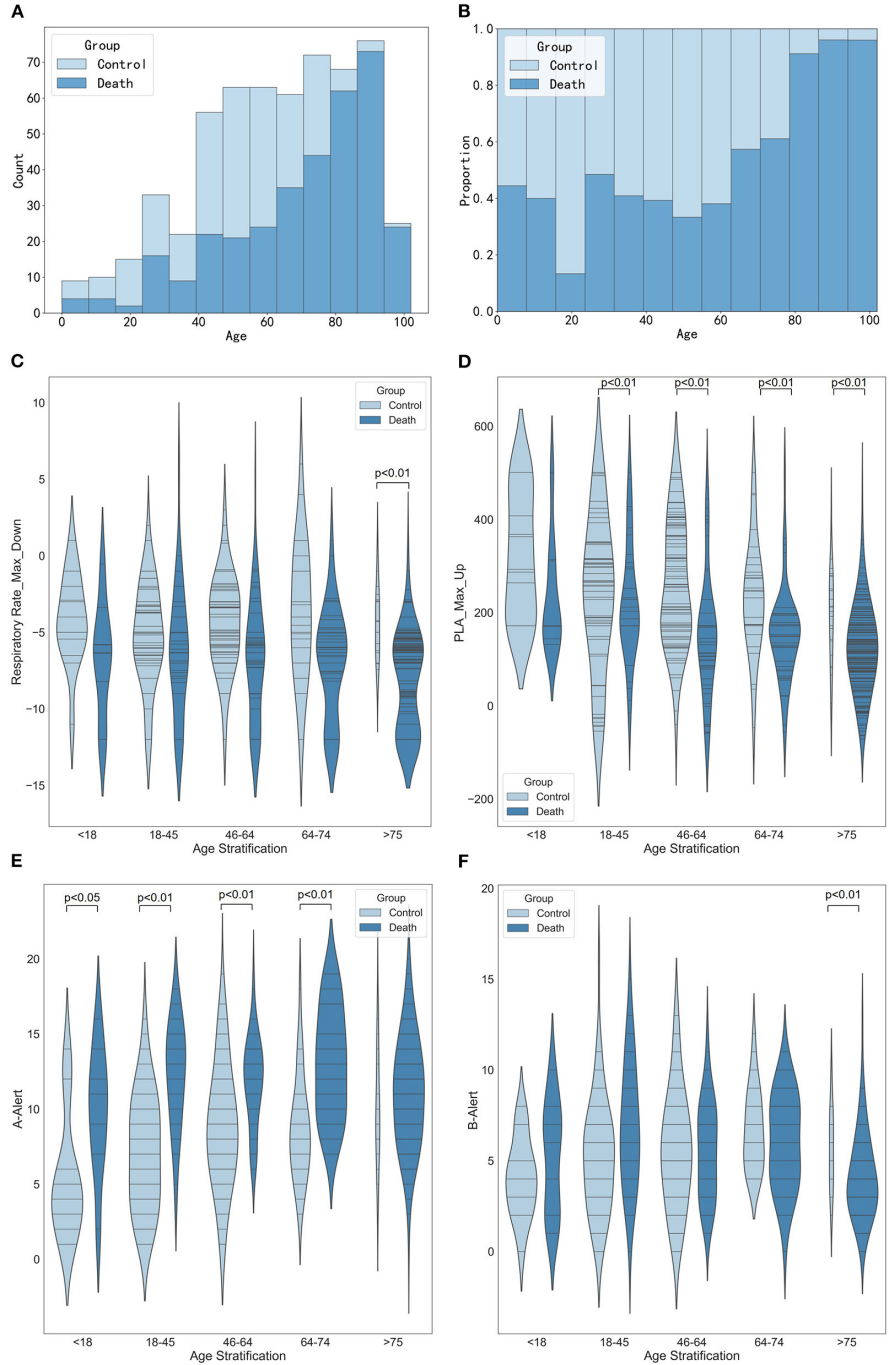
Stratified analysis of age. **(A)** Histogram showing the number of age stratification. **(B)** Histogram showing the proportion of age stratification. **(C)** Violin plots showing the maximum decrease in respiratory rate between different age stratification. **(D)** Violin plots showing the maximum increase in platelets between different age stratification. **(E)** Violin plots showing the number of A-alert drugs between different age stratification. **(F)** Violin plots showing the number of B-alert drugs between different age stratification.

These results in Figures 6A,B suggested an increasing trend in the number of deaths with increasing age. When the patients were older than 75, the maximum decrease in respiratory rate in the death group was significantly different from that in the control group (Figure 6C, *p* < 0.01). Figure 6D shows that when patients were older than 18, the maximum increase in platelets was significantly lower in the death group than that in the control group (all *p* < 0.01). Figure 6E shows that the older the age, the greater the number of A-alert drugs was used. And when the patients were younger than 75, the number of A-alert drugs used in the death group was significantly different from the control group (all *p* < 0.05 or *p* < 0.01). While the patients were older than 75, the number of B-alert drugs used in the death group was significantly different from that of the control group (Figure 6F, *p* < 0.01).

#### Stratified analysis of drug varieties

**Figure 7:**
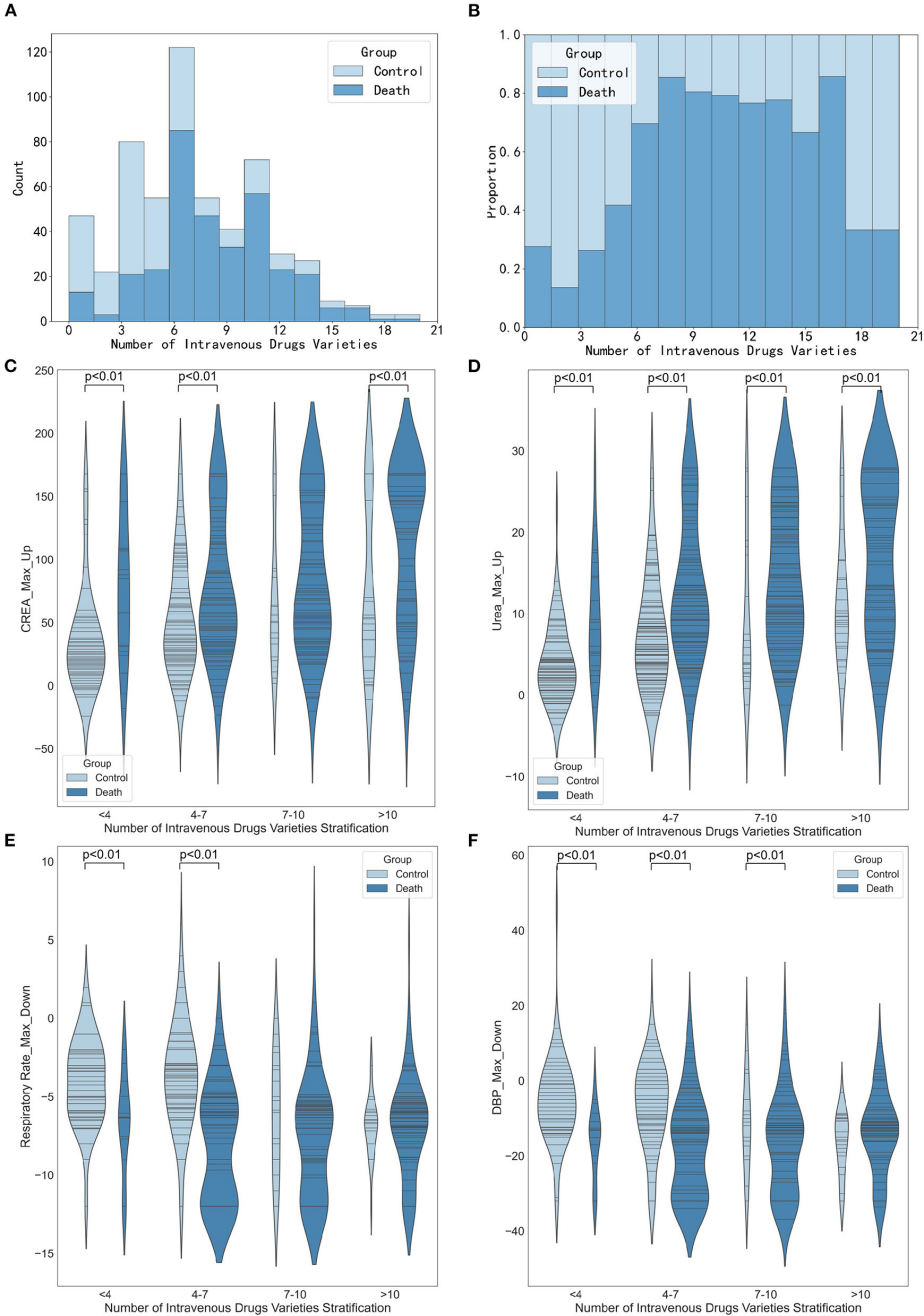
Stratified analysis of intravenous drugs varieties. **(A)** Histogram showing the number of intravenous drugs varieties stratification. **(B)** Histogram showing the proportion of intravenous drugs varieties stratification. **(C)** Violin plots showing the maximum increase in creatinine between different intravenous drugs varieties stratification. **(D)** Violin plots showing the number of maximum increase in urea between different intravenous drugs varieties stratification. **(E)** Violin plots showing the maximum decrease in respiratory rate between different medication varieties stratification. **(F)** Violin plots showing the maximum decrease in diastolic diastolic blood pressure between different medication varieties stratification.

Figure 7 shows the association between the number of drug varieties and the maximum increase in creatinine, the maximum increase in urea, the maximum decrease in respiratory rate, and the maximum decrease in diastolic blood pressure. The results in Figures 7C,D show that the higher the number of intravenous drug varieties, the more significant the maximum increase was in creatinine and urea (*p* < 0.01 or *p* < 0.05). Figure 7E reveals the association between the number of intravenous drug varieties and the maximum decrease of respiratory rate. When the number of intravenous drug varieties was <7, the maximum decrease of respiratory rate in the death group was significantly smaller than that of the control group (*p* < 0.01). Figure 7F suggests that when the number of intravenous drug varieties was <10, the maximum decrease in diastolic blood pressure in the death group was statistically significantly different from that of the control group (all *p* < 0.01), and close attention should be paid to patients whose maximum decreases in diastolic blood pressure were small and the number of intravenous drug varieties was more than 10.

## Discussion

P. aeruginosa infection constitutes a major clinical challenge (29). Therefore, it is of great significance to predict the risk factors of mortality for P. aeruginosa in severe patients. In this study, we assessed the risk factors of 571 patients with severe infection with P. aeruginosa, such as 338 deaths and 233 cures. A prediction model for mortality risk of P. aeruginosa in severe patients was established. Compared to some other machine learning algorithms, the XGBoost model achieved the best performance in ACC, AUROC, and AUPRC. Furthermore, in order to indicate the relationship between the clinical variables and the risk of mortality, the SHAP values were introduced to evaluated the importance of clinical variables in predictor.

The most obvious finding to emerge from the analysis above was that advanced age was one of the mortality risk factors for P. aeruginosa infection in severe patients, which was consistent with the results of a previous study (10). Then, the number of high-alert medication types and intravenous drug types were risk factors for mortality from P. aeruginosa infection, which had not been described in previous severe infection studies. The combination of many drugs is likely to cause some side effects on patients, and studies have shown that paying attention to high-risk drugs will greatly reduce hospitalization, disability, death, and other conditions (30). When the number of high-alert medication types and intravenous drugs types is too much, it reminds clinicians to pay more attention to the medication situation of patients. Timely adjustments of medication regimen are expected to improve the prognosis and reduce mortality of patients. Drainage is also a mortality risk factor for P. aeruginosa infection in severe patients. The results of this study indicate that patients who have been drained have a lower risk of mortality than patients who do not have been drained. This result is in accord with the fact that drainage is conducive to the timely discharge of purulent secretions, effusions, blood, and exudates from the wound. Drainage might possess dual roles in clinical treatment, one in assessing the condition patients and the other in facilitating wound healing. For abscess without effective drainage, the minimum effective concentrations for antimicrobial activity may not be reached.

In addition, we further stratified to explore the relationships between the site of infection, age stratification, and the number of medication species with important variables, laying the foundation for future variable interaction studies. The conclusion also verified that the risk of death from blood culture with P. aeruginosa was higher than other sites, and it was consistent with our common knowledge. Simultaneously, with patients' ages increasing and the higher the number of intravenous drug varieties used, the number of deaths showed an increasing trend. It suggested that risk factors such as advanced age and the number of drug varieties used need to be actively paid attention to for patients infected with P. aeruginosa, especially when the age was greater than 75 and the number of drug species was > 10. These conclusions were preliminary and needed to be further validated.

This retrospective study still had several limitations. Firstly, this was a single-center study and, therefore, has all the limitations inherent in such a study design. The distribution and characteristics of the clinical data used in this study could vary among different regions. In future works, integrating more data and having more precise estimates are possible. The clinical data from the multi-centric will help researchers to build more generalization and prospective prediction models. Future works should be used in elucidate the diversity of AMP resistance mechanisms in more realistic clinical settings. In future works, our model should be used in the multi-centric study or other clinical datasets such as MIMIC III (31) or a critical care database involving patients with infection (32). Secondly, our model was built based on the clinical data from patients with P. aeruginosa infection. Although it showed some predictive capacity in testing on clinical data from patients with Klebsiella pneumoniae infection, it still has significant shortcomings compared to the performance in P. aeruginosa infection. And it needs to be improved in future studies to expand the application of the model. Thirdly, the clinical data used in this study only included part of structured clinical information. Other informative data, such as nursing notes and radiology reports were not used. More detailed clinical data such as drug dosage or time, mechanical ventilation time and effectiveness evaluation will provide a new perspective for deep analysis and interpretation. Finally, since only the correlation rather than the causal relationship between the predictors and risk outcome was considered in this study, our conclusions still required further prospective trials to evaluate. More in-depth investigation of the causal relationship between the clinical feature and risk is essential for supporting clinical control and decision.

## Data availability statement

The original contributions presented in the study are included in the article/supplementary material, further inquiries can be directed to the corresponding authors.

## Ethics statement

The studies involving human participants were reviewed and approved by Institutional Medical Ethical Committee of the First affiliated Hospital of Air Force Medical University, China (approval No. KY20212130-C-1) on August 30, 2021. Written informed consent for participation was not required for this study in accordance with the national legislation and the institutional requirements.

## Author contributions

MW and XZ contributed to data collecting and processing. CC contributed to modeling and calculations. FM and MT performed the results analysis. CC, FM, and MT drafted the manuscript. RL contributed to manuscript revision. JW and YG provided overall supervision and undertook the responsibility of submitting the manuscript for publication. All authors discussed and commented on the manuscript.

## Funding

This research was financially supported by the National Natural Science Foundation of China (Nos. 72074218, 81903837, and 81774190).

## Conflict of interest

The authors declare that the research was conducted in the absence of any commercial or financial relationships that could be construed as a potential conflict of interest.

## Publisher's note

All claims expressed in this article are solely those of the authors and do not necessarily represent those of their affiliated organizations, or those of the publisher, the editors and the reviewers. Any product that may be evaluated in this article, or claim that may be made by its manufacturer, is not guaranteed or endorsed by the publisher.
