# Supplementary material for: A prediction and interpretation machine learning framework of mortality risk among severe infection patients with pseudomonas aeruginosa
Source: Front Med (Lausanne). 2022 Jul 25;9:942356. doi: 10.3389/fmed.2022.942356 (PMC9358029; doi:10.3389/fmed.2022.942356)
Supplement: Supplementary file 4 [file Table_4.DOCX]

**Table S4.** Hyperparameters space considered for LightGBM

| Hyperparameter | Values considered |
| --- | --- |
| The number of leaves | 5, 10, 15, 20, 25, 30, 35, 40 |
| Learning rate | 0.01, 0.05, 0.1, 0.2, 0.3, 0.5 |
| The number of estimators | 2, 4, 8, 16, 32, 64 |

**Table S5.** Hyperparameters space considered for CatBoost

| Hyperparameter | Values considered |
| --- | --- |
| Learning rate | **0.01, 0.05, 0.1, 0.2, 0.5, 1** |
| The max depth | **2, 4, 6, 8, 10, 16** |

**Table S6.** Hyperparameters space considered for Support Vector Machines

| Hyperparameter | Values considered |
| --- | --- |
| Regularization parameter C | 0.01, 1, 10, 100 |
| Kernel coefficient | 0.01, 1, 10 |
| Kernel type | 'linear', 'sigmoid', 'rbf', 'poly' |

**Table S7.** Hyperparameters space considered for Random Forests

| Hyperparameter | Values considered |
| --- | --- |
| The number of features | 'sqrt', 'log2' |
| The maximum depth | 4, 8, 16, 32, |
| Criterion | 'entropy', 'gini' |
| The number of trees | 2, 4, 6, 8 |
